# Quick Minds Slowed Down: Effects of Rotation and Stimulus Category on the Attentional Blink

**DOI:** 10.1371/journal.pone.0013509

**Published:** 2010-10-21

**Authors:** Sander Martens, Ozlem Korucuoglu, Henderikus G. O. M. Smid, Mark R. Nieuwenstein

**Affiliations:** 1 Neuroimaging Center, University Medical Center Groningen, University of Groningen, Groningen, The Netherlands; 2 Psychiatry Department, University Medical Center Groningen, Groningen, The Netherlands; 3 Experimental and Work Psychology, University of Groningen, Groningen, The Netherlands; University of Sydney, Australia

## Abstract

**Background:**

Most people show a remarkable deficit to report the second of two targets when presented in close temporal succession, reflecting an attentional restriction known as the ‘attentional blink’ (AB). However, there are large individual differences in the magnitude of the effect, with some people showing no such attentional restrictions.

**Methodology/Principal Findings:**

Here we present behavioral and electrophysiological evidence suggesting that these ‘non-blinkers’ can use alphanumeric category information to select targets at an early processing stage. When such information was unavailable and target selection could only be based on information that is processed relatively late (rotation), even non-blinkers show a substantial AB. Electrophysiologically, in non-blinkers this resulted in enhanced distractor-related prefrontal brain activity, as well as delayed target-related occipito-parietal activity (P3).

**Conclusion/Significance:**

These findings shed new light on possible strategic mechanisms that may underlie individual differences in AB magnitude and provide intriguing clues as to how temporal restrictions as reflected in the AB can be overcome.

## Introduction

Ranging from the Olympic Winter Games, bankers' bonuses, to student exams, individual differences in human performance play a pivotal role in (Western) society. Despite the fact that variability in performance can have profound consequences in daily life (e.g., traffic accidents), it is an aspect that has long been ignored in research on the attentional blink; a phenomenon that for the past two decades has been central in the field of temporal attention research [1].

The attentional blink (AB) is a deficit in reporting the second of two targets when presented within 200–500 ms after the first target [2]. Typically, participants are required to report two unspecified letters (the targets) among a rapid stream of sequentially presented digits (the non-targets or distractors). Although alphanumeric stimuli are most commonly used, the effect is very robust and can be obtained in the majority of people using a variety of stimuli and task conditions. Because semantic processing of unreported targets seems to be largely unaffected during an AB [3], [4], [5], [6], the effect is thought to reflect a very general property of perceptual awareness with broad implications for understanding how the brain perceives any task-relevant stimulus.

Whereas limited resources of some sort have been ascribed an important role in the AB [7], [8], a more complex picture has suddenly emerged from recent behavioral studies as well as from computational simulations, suggesting that attentional control [1], [9], [10], [11], [12], [13], [14], [15] and a tradeoff between identity and episodic forms of information is involved [16]. That is, rather than a lack of attentional capacity to process or consolidate the targets, there seems to be a protection process that temporarily inhibits or delays the processing of subsequent stimuli. This is assumed to minimize interference with T1 while it is being consolidated in working memory, but comes at a cost for T2, as reflected in the AB. Given that distraction by task-irrelevant stimuli [17], [18] or even a concurrent secondary task [11], [19] can attenuate the magnitude of the AB effect, it has been argued that this protection is no longer needed when attention is distributed more optimally. These recent findings have dramatically changed the theoretical landscape, resulting in a vibrant and as of yet unsettled debate.

Adding to the debate and germane to the current paper, we have recently shown that there are large individual differences in AB magnitude, and that in some individuals, (about 5% of the population), the AB is absent altogether in a task that requires identification of two letter targets embedded amongst digit distractors [20]. Even when the stimulus duration is decreased substantially, these so-called ‘non-blinkers’ show a remarkable ability to successfully identify both targets, regardless of the time interval or lag between the targets [20], [21], thereby questioning the fundamental nature of the AB phenomenon.

In comparison to regular ‘blinkers’ (individuals who do show an AB), it has been found that the non-blinkers neither seem to differ in short-term memory capacity, working memory capacity, nor in general intelligence level [22]. In contrast, however, EEG measurements have revealed differences in parietal and frontal brain activity, reflecting differences in target processing [20]. More target-related activity was found over the ventrolateral prefrontal cortex (assumed to play a role in a wide range of cognitive processes, including the selection of nonspatial information), whereas blinkers showed more distractor-related prefrontal activity. These findings suggest that non-blinkers are more efficient in distinguishing targets from distractors at a relatively early processing stage. Converging evidence from behavioral studies confirmed that non-blinkers are better in ignoring distractors than blinkers are [23], [24]. Finally, regardless of the lag between the targets, non-blinkers were found to be quicker in consolidating the identity of targets than blinkers, reflected in the latencies of the P3 ERP components (associated with working memory updating) induced by successfully identified targets [20].

Given these findings, it has been suggested that a major source of individual variability in AB magnitude may lie in processes of selective attention that are involved in determining which objects are selected for further processing and memory consolidation [22]. In other words, the occurrence of an AB may be determined by an allocation policy, which might vary from individual to individual. An efficient early selection strategy should be rendered more difficult or even impossible, if targets and distractors become harder to distinguish and identify [25]. The aim of the current study, consisting of two behavioral experiments and one EEG experiment, was to test this. Rather than visually degrading the stimuli, target selection difficulty was manipulated by rotation of the targets and/or the distractors, thus keeping the visual quality of the stimuli intact, but rendering selection of targets a more time-consuming process [11], [26], [27]. It was predicted that under such experimental conditions even non-blinkers should show an AB.

## Methods

### Experiment 1

#### Participants

On the basis of previous performance in AB experiments in our laboratory in which two targets had to be identified among an RSVP stream of distractors [12], [20], [21], [22], [24], [28], [29], two groups of volunteers were formed: A blinker group (seven female, aged 21–35, mean 24.5) and a non-blinker group (seven female, aged 21–27, mean 23.6), consisting of 12 participants each. Similar to [20], a participant was considered to be either a non-blinker or blinker when AB magnitude (the percentage of decrement in T2 performance within the AB period relative to T1 performance) had consistently been either smaller or larger than 15%, respectively. The selected non-blinkers had a mean AB magnitude of 3.9% (range  = −4.2 to 12.2%), whereas blinkers had a mean AB magnitude of 34.3% (range  = 16.5 to 74.7%). All participants were recruited from the University of Groningen community and had normal or corrected-to-normal visual acuity. The Neuroimaging Center Institutional Review Board approved the experimental protocol and written consent was obtained prior to the experiment. Participants received payment of 10 €.

#### Stimuli and apparatus

The generation of stimuli and the collection of responses were controlled using E-prime 1.2 software [30] running under Windows XP on a PC with a 2.8 Ghz processor. Stimuli consisted of the digits 2, 3, 4, and 5 and uppercase letters (excluding C, H, I, M, N, O, Q, S, U, W, X, Y, Z due to their similarity with the rotated versions of other letters or being identical to the rotated version of themselves) and were presented in black (2 cd/m^2^) on a white background (88 cd/m^2^) in a 18-point Courier New font on a 17-in. CRT monitor with a 100-Hz refresh rate. The stimuli subtended ∼1° by 1° of visual angle at a viewing distance of approximately 60 cm.

#### Procedure

**Figure 1:**
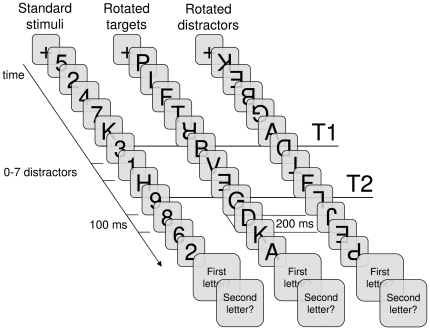
The AB paradigm. Schematic representation of the AB paradigm as used in Experiment 1 with standard stimuli, rotated targets, or rotated distractors, respectively.

The experiment consisted of three conditions: A standard AB condition, a rotated targets condition, and a rotated distractors condition (see Figure 1).

In the standard AB condition, participants were asked to identify two letter targets (T1 and T2) presented within a rapid serial visual presentation (RSVP) stream of 13 digit distractors. Before each trial, a message was presented at the bottom of the screen, prompting participants to press the space bar to initiate the trial. When the space bar was pressed the message disappeared immediately. After 250 ms, a fixation cross appeared in the middle of the screen for 500 msec, followed 100 ms later by the RSVP stream, consisting of 15 sequentially presented items.

Distractors were presented for 100 ms. On the first trial of each block, targets were presented for 90 ms, immediately followed by a 10-ms mask (a digit; for simplicity reasons masks are not shown in Figure 1). We attempted to control overall condition difficulty, keeping mean T1 performance at approximately 85%, by manipulating the duration of both targets in the following way. After the first trial, target and mask duration were variable, with target duration ranging from 20 to 90 ms. The sum of target and mask duration was always 100 ms, thereby keeping the interval between the onset of a target and the onset of a subsequent stimulus constant. After each trial, a running average of T1 accuracy was calculated. Whenever mean T1 accuracy became higher than 90%, target presentation duration was decreased by 10 ms and mask duration was increased by 10 ms, thereby making T1 identification more difficult. When mean T1 accuracy dropped below 80%, target presentation duration was increased by 10 ms and mask duration decreased by 10 ms, thereby making T1 detection easier.

T1 was always presented as the fifth item in the stream. T2 varied from being the first (lag 1) to the eighth item (lag 8) after T1, and was always followed by at least two additional distractors. Target letters were randomly selected with the constraint that T1 and T2 were always different letters. Digit distractors and masks were randomly selected with the constraint that no single digit was presented twice in succession. After the stream was presented, participants were prompted by a message at the bottom of the screen to type the letters they had seen using the corresponding keys on the computer keyboard. Participants were instructed to take sufficient time in making their responses to ensure that typing errors were not made. Participants were encouraged to type in their responses in the order in which the letters had been presented, but responses were accepted and counted correct in either order.

The rotated targets condition was the same as the standard AB condition except for the following changes. All stimuli consisted of letters only, and targets differed from distractors by having been rotated 180 degrees (clockwise). Participants were instructed to report the two rotated letters. As this letters-only condition was much more difficult, the duration of each unrotated distractor letter was increased to 200 ms, as well as the total duration of a target and its immediate mask. Initial target duration was 190 ms, immediately followed by a 10 ms mask (an unrotated letter). After the first trial, target and mask duration were variable using the same running-average procedure as in the standard AB condition, but with target duration ranging from 20 to 190 ms.

The rotated distractors condition was the same as the rotated targets condition, the only difference being that now the distractors consisted of rotated letters whereas the targets were unrotated letters.

The experiment always started with the standard AB condition, in order to retest each individual's AB magnitude, ensuring that the previously observed lack or presence of a sizable AB effect was consistent across experiments and testing sessions. The order of the other two conditions was counterbalanced across subjects. Each condition included one practice block consisting of 24 trials, and three testing blocks of 64 trials each, such that each combination of condition and SOA was repeated 24 times. After each block, a short break was given with a somewhat longer break after each condition. Participants completed the experiment in approximately 90 minutes.

### Experiment 2

To determine whether rotation or the lack of alphanumeric category information caused the non-blinkers to blink in Experiment 1, similar conditions were used as in Experiment 2, but apart from being rotated or not, targets and distractors could be distinguished on the basis of their stimulus category. As in the standard AB condition, targets always consisted of a letter that had to be identified, whereas distractors consisted of an irrelevant digit. If the lack of alphanumeric category information was the main cause of the non-blinkers' AB in Experiment 1, rather than rotation or a combination of both factors, little or no AB should occur for non-blinkers in Experiment 2, in which category information was present in all conditions.

#### Participants

Except for three blinkers, all participants from Experiment 1 volunteered to participate in Experiment 2. The three blinkers were replaced by three new participants (aged 23–28), who had normal or corrected-to-normal visual acuity, and were recruited from the University of Groningen community. Prior to their participation in Experiment 2, new participants were tested using the standard AB condition from Experiment 1, thereby assuring that they were indeed blinkers. The Neuroimaging Center Institutional Review Board approved the experimental protocol and written consent was obtained prior to the experiment. Participants received payment of 10 €.

#### Stimuli and apparatus

Stimuli and apparatus were the same as in Experiment 1.

#### Procedure

The experiment consisted of three conditions: A rotated targets condition, a rotated distractors condition, and a condition in which all stimuli were rotated. In all conditions, targets consisted of letters, whereas distractors consisted of digits. The procedure was the same as in Experiment 1, except for the following changes. In all conditions, distractors were presented for 100 ms each. Each block of trials began with a target duration of 70 ms, immediately followed by a 30-ms masking digit. After the first trial, target and mask duration were manipulated as in Experiment 1, but with target duration ranging from 20 to 90 ms and mask duration ranging from 80 to 10 ms. The SOA between targets was identical in all three conditions, ranging from 100 to 800 ms (lags 1–8). The order of conditions was counterbalanced across participants. The experiment took approximately 90 minutes to complete.

### Experiment 3

Given the findings from Experiment 1 and 2, we predict that when category information does not distinguish targets from distractors, non-blinkers are forced to process each stimulus much more elaborately, rendering an efficient selection of targets difficult or impossible. In Experiment 3, we adapted Experiment 1 to include EEG recordings, to see whether the absence of category information indeed leads to an increase in brain activity in response to each distractor, reflecting more elaborate processing.

#### Participants

On the basis of previous performance in AB experiments in our laboratory in which two targets had to be identified among an RSVP stream of distractors, a group of 10 new blinkers (six female, aged 21–20, mean 24.5) and a group of 9 non-blinkers (seven female, aged 18–26, mean 22.7, of whom 7 had participated in the previous two experiments) were formed. All participants were recruited from the University of Groningen community and had normal or corrected-to-normal visual acuity. The Neuroimaging Center Institutional Review Board approved the experimental protocol and written consent was obtained prior to the experiment. Participants received payment of 20 .

#### Stimuli and Apparatus

Stimuli and apparatus were the same as in Experiment 1.

#### Procedure

The procedure and conditions were the same as in Experiment 1, with the following exceptions.

In a third of the trials, no targets were presented (no-target trials), only distractors. Participants were informed that some trials would not include any targets. At the end of such no-target trials, participants were to indicate the absence of targets by pressing the space bar twice.

In two-thirds of the trials, two targets were presented (dual-target trials) within the stream of distractors. When targets were present, T1 was always presented as the fifth item in the stream. In the standard AB condition, T2 was either the fourth (lag 4) or the tenth item (lag 10) following T1, yielding SOAs of 400 and 1000 ms, respectively. T2 was always followed by at least seven additional distractors. In the letters-only conditions (with either the rotated targets or rotated distractors), T2 was either the second (lag 2) or the fifth item (lag 5) following T1, yielding SOAs of 400 and 1000 ms, respectively. T2 was always followed by at least four additional distractors.

Each condition included one practice block consisting of 9 trials, and three testing blocks of 72 trials each, such that each combination of condition, trial type (two targets or no targets) and SOA was repeated 72 times. After each block, a short break was given with a somewhat longer break after each condition. Participants completed the experiment in approximately 2 hours.

#### EEG recording

The EEG signal was recorded using a 64-channel electro-cap with tin electrodes. The electro-cap was organized according to the international 10/20 system and connected to an REFA 8–64 average reference amplifier. Impedance was reduced to less than 5 kΩ for all electrodes. Data was sampled with a frequency of 2 kHz and digitally reduced to 250 Hz. Two electrodes connected to the mastoids served as an offline reference. The horizontal electrooculogram (EOG) was recorded from tin electrodes attached approximately 1 to 2 cm to the left and right of the outside corner of each eye. The vertical EOG was recorded from two tin electrodes attached approximately 3 cm below the left eye and 1 cm above the brow of the left eye, respectively. Brain Vision Recorder 1.10 software (Brain Products GmbH, Munich, Germany) was used to control the data acquisition.

#### Data analysis

The data were analyzed by using Brain Vision Analyzer 1.05 software (Brain Products). The ERPs were time locked to the onset of the RSVP stream, had a duration of 2200 ms, and were calculated relative to a 200-ms prestream baseline, yielding a total length of 2400 ms. The ERP-segments were 20-Hz low-pass filtered, corrected for eye movements, DC detrended (to remove direct current drift artifacts), and baseline corrected before artifact rejection was applied. Segments with maximum differences of values greater than 100 µV (i.e., containing artifacts) were excluded from further analysis (a total of 7.2% of the trials, ranging from 0 to 21.3%, SD  = 6.46, of the trials per participant). When appropriate, Greenhouse-Geisser-corrected p values are reported.

## Results and Discussion

### Experiment 1

Where appropriate, Greenhouse-Geisser-corrected p values are reported. As the rate of presentation in the standard AB condition was different from that in the other conditions, performance in the standard AB condition was analyzed separately.

#### Target durations

In the standard AB condition, mean target duration was 67 ms for non-blinkers and 74 ms for blinkers, which, however, was not significantly different (p = .08). In the letters only conditions, mean target duration tended to be lower for non-blinkers (165 ms in the rotated targets condition and 180 ms in the rotated distractors condition) than for blinkers (171 ms in the rotated targets condition and 186 ms in the rotated distractors condition). However, a separate mixed analysis of variance (ANOVA) with group (non-blinkers or blinkers) as between-subjects factor and condition (rotated targets or rotated distractors) as within-subjects factor revealed a significant effect of condition, F(1, 22) = 34.82, MSE  = 71.41, p<.001, η^2^_p_ = .61, but no significant effect of group (p = .18) and no interaction (p = .87). These results suggest that the rotated distractors condition in particular was a challenging condition for both groups.

#### T1 performance

**Figure 2:**
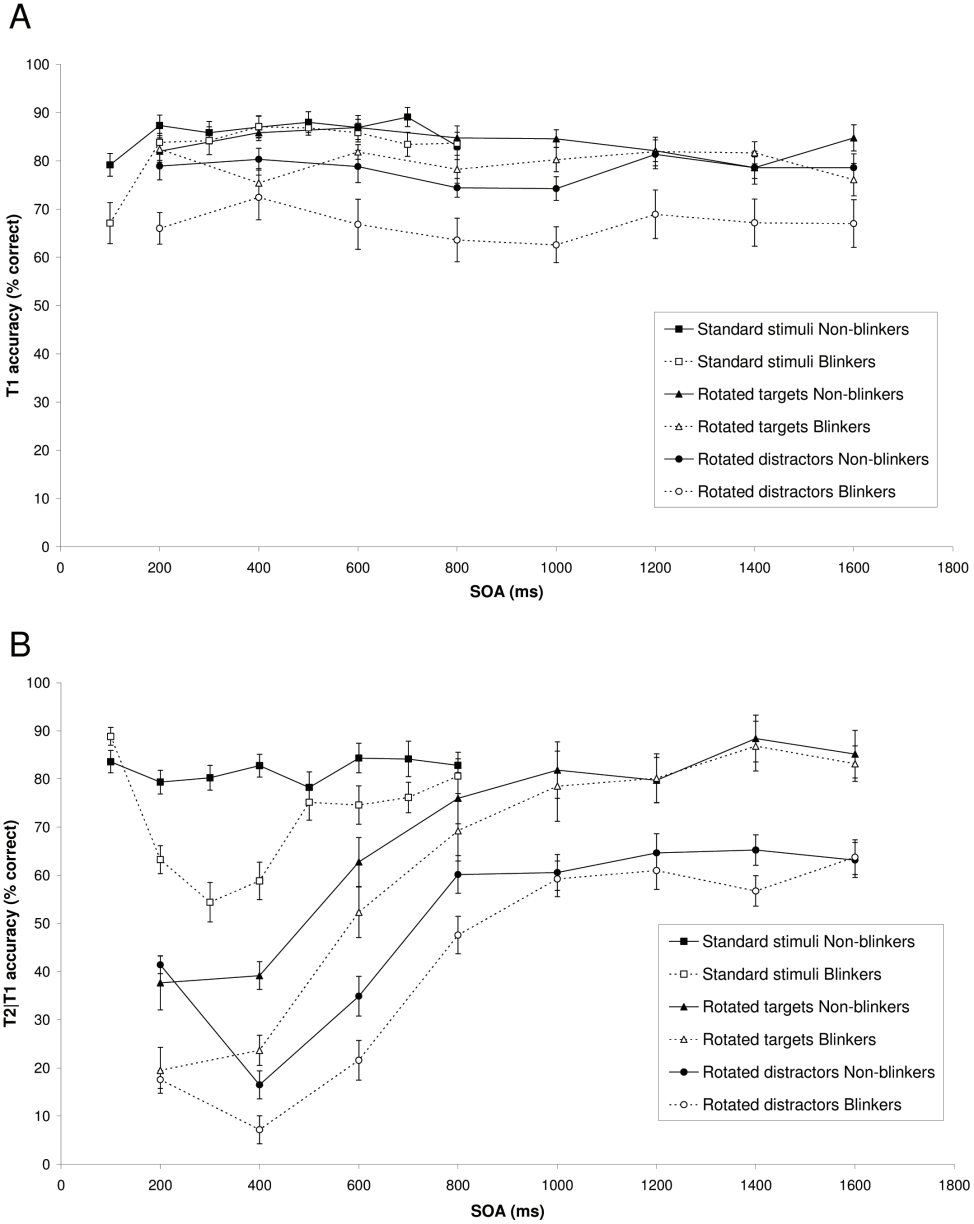
Target accuracy in Experiment 1. (A) Mean percentage correct report of T1 in the standard, rotated targets, and rotated distractors conditions of Experiment 1 as a function of target SOA, for non-blinkers and blinkers. (B) Mean percentage correct report of T2 in the standard, rotated targets, and rotated distractors conditions of Experiment 1, given correct report of T1, as a function of target SOA, for non-blinkers and blinkers. Error bars reflect standard error of the mean.

Despite our efforts to keep T1 performance similar across groups and conditions, significant differences in performance were found. Figure 2A shows T1 performance in the three conditions as a function of the stimulus onset asynchrony (SOA) between the targets for non-blinkers and blinkers, respectively. In the standard AB condition mean T1 performance was 85.8% for non-blinkers and 82.7% for blinkers. A mixed ANOVA with group (non-blinkers or blinkers) as a between-subjects factor and SOA (100 to 800 ms, corresponding to lags 1-8) as a within-subjects factor revealed a significant effect of group, F(1, 22) = 6.23, MSE  = 71.84, p = .02, η^2^_p_ = .22, reflecting non-blinkers to perform slightly better than blinkers did. A main effect of SOA was also found, F(7, 154) = 8.02, MSE  = 65.54, p<.001, η^2^_p_ = .27. Bonferroni-corrected pairwise comparisons showed that performance at SOA 100 (lag 1) was worse than at the other SOAs (ps<.01). No significant interaction between group and SOA was found (p = .17).

Mean T1 performance in the rotated targets condition was 83.7% for non-blinkers and 79.7% for blinkers. In the rotated distractors condition, mean T1 performance was 78.2% for non-blinkers and 66.8% for blinkers. A mixed ANOVA with group (non-blinkers or blinkers) as a between-subjects factor and condition (rotated targets or rotated distractors) and SOA (200 to 1600 ms, corresponding to lags 1-8) as within-subjects factors revealed significant effects of group, F(1, 22) = 7.95, MSE  = 707.29, p = .01, η^2^_p_ = .27, condition, F(1, 22) = 37.11, MSE  = 220.11, p<.001, η^2^_p_ = .63, and a significant Group × Condition interaction, F(1, 22) = 5.95, MSE  = 220.11, p = .02, η^2^_p_ = .21, reflecting that, specifically in the rotated distractors condition, the blinkers' T1 performance was worse than that of the non-blinkers. No significant effect of SOA or any other significant interactions were found (ps>.16).

#### T2 performance

Figure 2B shows T2 performance in the three conditions, given that T1 was identified correctly, as a function of SOA for non-blinkers and blinkers, respectively. For the standard AB condition, a mixed ANOVA with group (non-blinkers or blinkers) as a between-subjects factor and SOA (100–800 ms) as within-subjects factors revealed significant effects of group, F(1, 22) = 16.28, MSE  = 322.54, p<.001, η^2^_p_ = .43, and SOA, F(7, 154) = 10.69, MSE  = 92.04, p<.001, η^2^_p_ = .33. In addition, a significant Group × SOA interaction was found, F(7, 154) = 7.68, MSE  = 92.04, p<.001, η^2^_p_ = .26. A separate ANOVA for the non-blinkers revealed no effect of SOA (F<1), confirming that they show little or no AB effect.

For the letters only conditions, a mixed ANOVA with group (non-blinkers or blinkers) as a between-subjects factor and condition (rotated targets or rotated distractors) and SOA (200–1600 ms) as within-subjects factors revealed significant effects of group, F(1, 22) = 5.65, MSE  = 1109.47, p = .027, η^2^_p_ = .20, condition, F(1, 22) = 62.70, MSE  = 548.46, p<.001, η^2^_p_ = .74, and SOA, F(7, 154) = 96.51, MSE  = 228.73, p<.001, η^2^_p_ = .81. In addition, a significant Condition × SOA interaction was found, F(7, 154) = 7.39, MSE  = 132.03, p<.001, η^2^_p_ = .25. Figure 2B suggests that there was more lag-1 sparing in the rotated distractors condition than in the rotated targets condition. Other interactions were not significant, although the Group × SOA interaction was close to significance (p = .06). Separate pairwise comparisons suggested that the AB effect lasted at least 600 ms for both groups, as reflected in a significant drop in performance at SOAs 200–600 compared to longer SOAs (ps<.01).

Even though overall performance was better for non-blinkers than for blinkers, and the AB effect tended to be somewhat smaller for the non-blinkers, it is evident that both letters-only AB conditions led to a remarkably large AB effect, not only in blinkers, but also in non-blinkers. Although we were unable to keep T1 performance at the same level across groups and conditions, it is unlikely that the differences in T1 performance are (solely) responsible for the occurrence of an AB in non-blinkers, given that previous manipulations that negatively affected the non-blinkers' T1 performance did not lead to the occurrence of an AB [20], [21].

It remains unclear however whether the increased AB magnitude in both groups was due to the rotated stimuli, or was primarily caused by the fact that only letter stimuli were used. Experiment 2 was set up to clarify this and to test whether non-blinkers were able to make use of the alphanumeric category information that was present in the standard AB condition but absent in the rotated conditions. Non-blinkers may be highly efficient in distinguishing letter targets from digit distractors enabling selection at an early pre-bottleneck processing stage, thereby avoiding the occurrence of an AB.

### Experiment 2

#### Target durations

Performance in the three conditions was compared to that in the standard AB condition from Experiment 1 (including the data of the three new blinkers). For the non-blinkers, mean target durations were 67, 68, 67, and 68 ms for the standard stimuli, rotated targets, rotated distractors, and rotated stimuli condition, respectively. For the blinkers, mean target durations were 74, 71, 80, and 75 ms for the standard stimuli, rotated targets, rotated distractors, and rotated stimuli condition, respectively. An ANOVA on these target durations revealed a significant effect of group, F(1, 22) = 6.69, MSE  = 199.01, p = .02, η^2^_p_ = .23, reflecting blinkers to require longer target durations than non-blinkers did. The main effect of condition was marginally significant, F(3, 66)  = 2.71, MSE  = 27.31, p = .06, η^2^_p_ = .11, and a significant Group × Condition interaction was found, F(3, 66)  = 4.37, MSE  = 27.31, p = .01, η^2^_p_ = .17, reflecting the fact that especially blinkers required a relatively long target duration in the rotated distractors condition. It can be concluded that the conditions, especially the rotated distractors condition, were more difficult for the blinkers than for the non-blinkers. Due to our dynamic masking procedure though, a comparable level of T1 performance was obtained for both groups across the different conditions.

#### T1 performance

**Figure 3:**
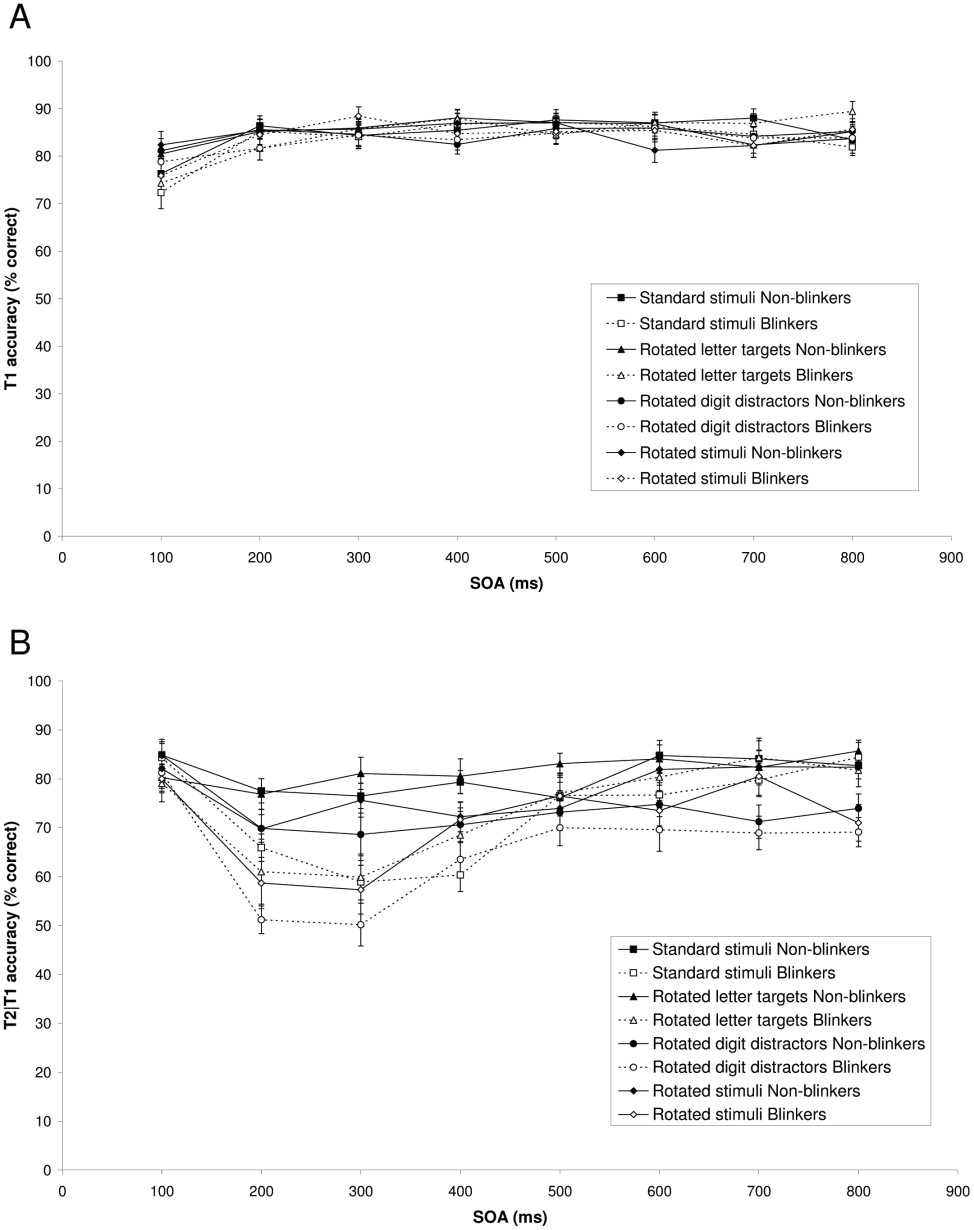
Target accuracy in Experiment 1 and 2. (A) Mean percentage correct report of T1 in the standard condition (Experiment 1), rotated targets, rotated distractors, and rotated stimuli conditions of Experiment 2 as a function of target SOA, for non-blinkers and blinkers. (B) Mean percentage correct report of T2 in the standard condition (Experiment 1), rotated targets, rotated distractors, and rotated stimuli conditions of Experiment 2, given correct report of T1, as a function of target SOA, for non-blinkers and blinkers. Error bars reflect standard error of the mean.

Figure 3A shows T1 performance in the four conditions as a function of the SOA between the targets for non-blinkers and blinkers, respectively. For the non-blinkers, mean T1 performance was 84.8% in the standard condition, 85.0% in the rotated targets condition, 84.4% in the rotated distractors condition, and 84.5% in the rotated stimuli condition. For the blinkers, mean T1 performance was 83.5% in the standard condition, 84.7% in the rotated targets condition, 83.4% in the rotated distractors condition, and 84.0% in the rotated stimuli condition. A mixed analysis of variance (ANOVA) with group (non-blinkers or blinkers) as a between-subjects factor and condition (standard stimuli, rotated targets, rotated distractors, or rotated stimuli) and SOA (100 to 800 ms, corresponding to lags 1–8) as a within-subjects factor revealed only a significant effect of SOA, F(7, 154)  = 10.04, MSE  = 60.61, p<.001, η^2^_p_ = .31. Pairwise comparisons showed that performance at SOA 100 (lag 1) was worse than at the other SOAs (ps<.01). Although there was a trend for a main effect of group (p = .07), neither condition (p = .24), nor any interactions (ps>.46) were significant, suggesting that T1 performance was largely comparable across groups and conditions.

#### T2 performance

Figure 3B shows T2 performance in the four conditions, given that T1 was identified correctly, as a function of SOA for non-blinkers and blinkers, respectively. A mixed ANOVA with group (non-blinkers or blinkers) as a between-subjects factor and condition (standard stimuli, rotated targets, rotated distractors, or rotated stimuli) and SOA (100–800 ms) as within-subjects factors revealed significant effects of group, F(1, 22)  = 19.20, MSE  = 741.70, p<.001, η^2^_p_ = .47, condition, F(3, 66)  = 12.30, MSE  = 227.57, p<.001, η^2^_p_ = .36, and SOA, F(7, 154) = 15.63, MSE = 197.19, p<.001, η^2^_p_ = .42. In addition, a significant Group × SOA interaction was found, F(7, 154)  = 6.54, MSE  = 197.19, p<.001, η^2^_p_ = .23, reflecting blinkers to show a larger AB than the non-blinkers did. In addition, a significant Condition × SOA interaction was found, F(21, 462)  = 2.06, MSE  = 81.60, p = .04, η^2^_p_ = .07, reflecting the AB to be the largest in the rotated distractors condition. The Group × Condition × SOA was not significant (p = .11). A separate pre-planned analysis for the non-blinkers revealed a significant effect of Condition, F(3, 33)  = 7.54, MSE  = 226.90, p<.001, η^2^_p_ = .41, but no significant effect of SOA (p = .07), and no significant interaction (p = .15), reflecting little or no AB effect. When only SOAs 200–800 were considered, an effect of Condition was still present, F(3, 33)  = 6.18, MSE  = 237.12, p = .002, η^2^_p_ = .36, but there was clearly no effect of SOA, (p = .16), and no interaction (p = .28) for non-blinkers. For the blinkers, a significant effect of Condition, F(3, 33)  = 5.10, MSE  = 228.23, p = .01, η^2^_p_ = .32, and SOA, F(7, 77)  = 15.20, MSE  = 265.08, p<.001, η^2^_p_ = .58, were found, but no significant interaction (p = .08), reflecting overall performance (across SOAs) in the rotated distractors condition to be worse than in the other conditions.

These results show that it was the lack of alphanumeric category information rather than rotation that caused the non-blinkers to blink in Experiment 1. For both groups, rotation did affect overall performance but did not seem to alter the magnitude or duration of the AB.

### Experiment 3

#### Target durations

In the standard stimuli condition, non-blinkers had a significantly shorter mean target duration (61.9 ms) than blinkers (75.9 ms), t(17) = 2.24, SD = 6.27, p = .04. For the non-blinkers, mean target duration was 137.8 ms in the rotated targets condition and 168.6 ms in the rotated distractors condition. For the blinkers, mean target duration was 163.6 ms in the rotated targets condition and 185.6 ms in the rotated distractors condition. A separate mixed ANOVA with group (non-blinkers or blinkers) as between-subjects factor and condition (rotated targets or rotated distractors) as within-subjects factor revealed a significant effect of group, F(1, 17)  = 10.06, MSE  = 432.69, p = .006, η^2^_p_ = .37, such that the mean target duration was shorter for the non-blinkers than for the blinkers. In addition, a significant effect of condition was found, F(1, 17)  = 39.45, MSE  = 167.22, p<.001, η^2^_p_  = .70, such that the mean target duration in the rotated target condition was shorter than in the rotated distractors condition. No significant interaction was observed (p = .31).

#### T1 performance

**Figure 4:**
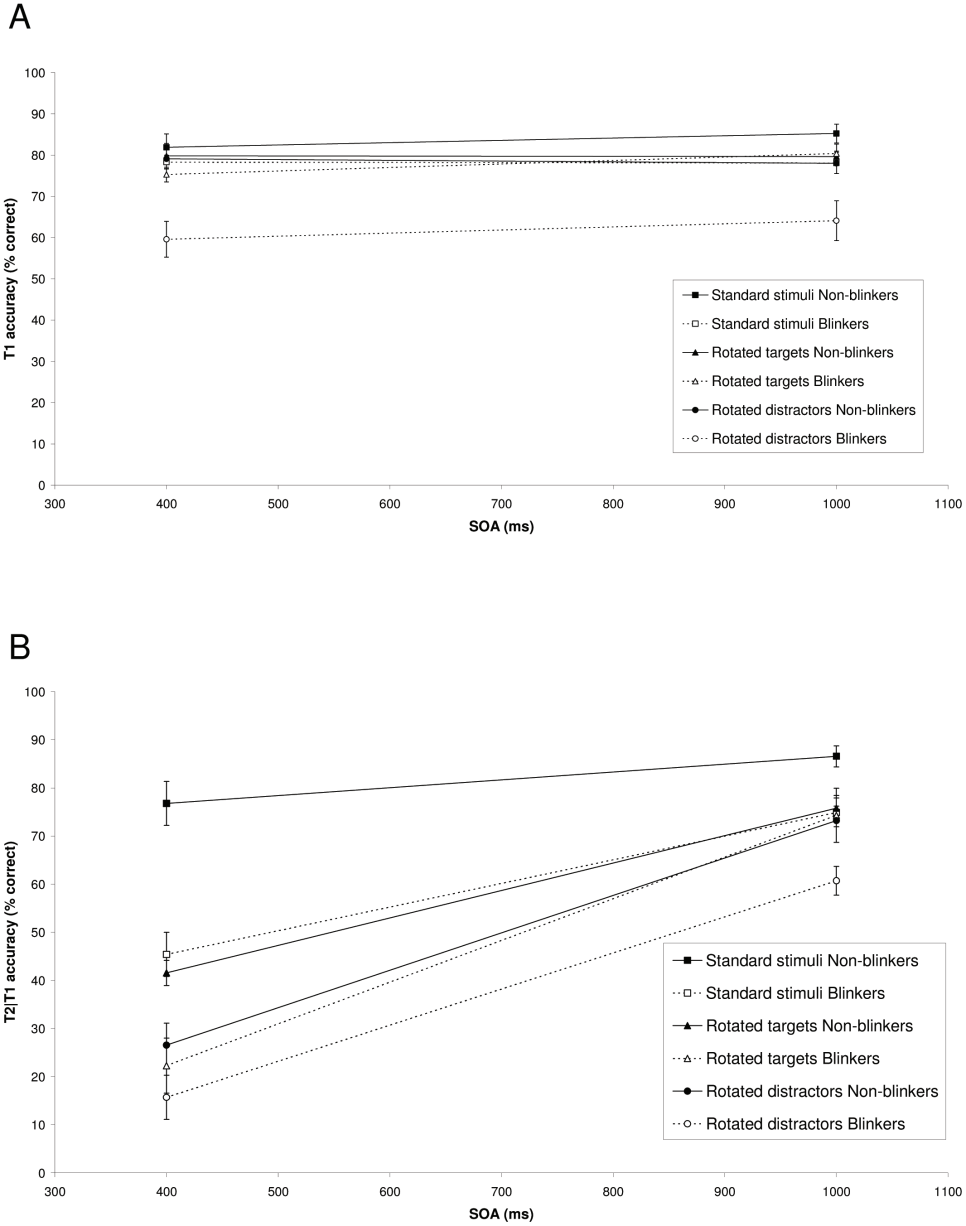
Target accuracy in Experiment 3. (A) Mean percentage correct report of T1 in the standard, rotated targets, and rotated distractors conditions of Experiment 3 as a function of target SOA, for non-blinkers and blinkers. (B) Mean percentage correct report of T2 in the standard, rotated targets, and rotated distractors conditions of Experiment 3, given correct report of T1, as a function of target SOA, for non-blinkers and blinkers. Error bars reflect standard error of the mean.

Figure 4A shows T1 performance in the three conditions as a function of SOA between the targets for non-blinkers and blinkers, respectively. For the non-blinkers, mean T1 performance was 84.2% in the standard condition, 84.0% in the rotated targets condition, and 82.7% in the rotated distractors condition. For the blinkers, mean T1 performance was 83.0% in the standard condition, 83.9% in the rotated targets condition, and 70.9% in the rotated distractors condition. A mixed ANOVA with group (non-blinkers or blinkers) as a between-subjects factor and condition (standard stimuli, rotated targets, or rotated distractors) and SOA (400 or 1000 ms) as within-subjects factors revealed a significant effect of group, F(1, 17) = 10.05, MSE  = 180.06, p = .006, η^2^_p_ = .37, condition, F(2, 34) = 12.24, MSE  = 99.07, p<.001, η^2^_p_ = .42, and a small but significant main effect of SOA, F(1, 17) = 4.87, MSE  = 21.48, p = .04, η^2^_p_ = .22, such that performance was slightly higher at the long SOA (77.6%) than at the short SOA (75.7%). Only the Group × Condition interaction reached significance, F(2, 34) = 5.75, MSE  = 99.07, p = .012, η^2^_p_ = .25, reflecting that, specifically in the rotated distractors condition, the blinkers' T1 performance was worse than that of the non-blinkers.

#### T2 performance

Figure 4B shows T2 performance in the three conditions, given that T1 was identified correctly, as a function of SOA for non-blinkers and blinkers, respectively. A mixed ANOVA with group (non-blinkers or blinkers) as a between-subjects factor and condition (standard stimuli, rotated targets, or rotated distractors) and SOA (400 or 1000 ms) as within-subjects factors revealed significant effects of group, F(1, 17) = 26.10, MSE  = 229.73, p<.001, η^2^_p_ = .61, condition, F(2, 34) = 36.99, MSE  = 190.29, p<.001, η^2^_p_ = .69, and SOA, F(1, 17) = 170.08, MSE  = 218.91, p<.001, η^2^_p_ = .91. In addition, a significant Group × SOA interaction was found, F(1, 17) = 4.63, MSE  = 218.91, p = .046, η^2^_p_ = .21, a Condition × SOA interaction, F(2, 34) = 25.42, MSE  = 77.31, p<.001, η^2^_p_ = .60, and a Group × Condition × SOA interaction, F(2, 34) = 4.29, MSE  = 77.31, p = .02, η^2^_p_ = .20. The results indicate that non-blinkers performed better than the blinkers in all conditions, but showed a considerable AB in both letters only conditions, replicating the findings from Experiment 1. Note that the blinker's relatively low performance at an SOA of 1000 ms in the rotated distractors condition is probably largely due to the fact that their overall T1 performance was also lower in this condition than in the other conditions.

#### The P3

**Figure 5:**
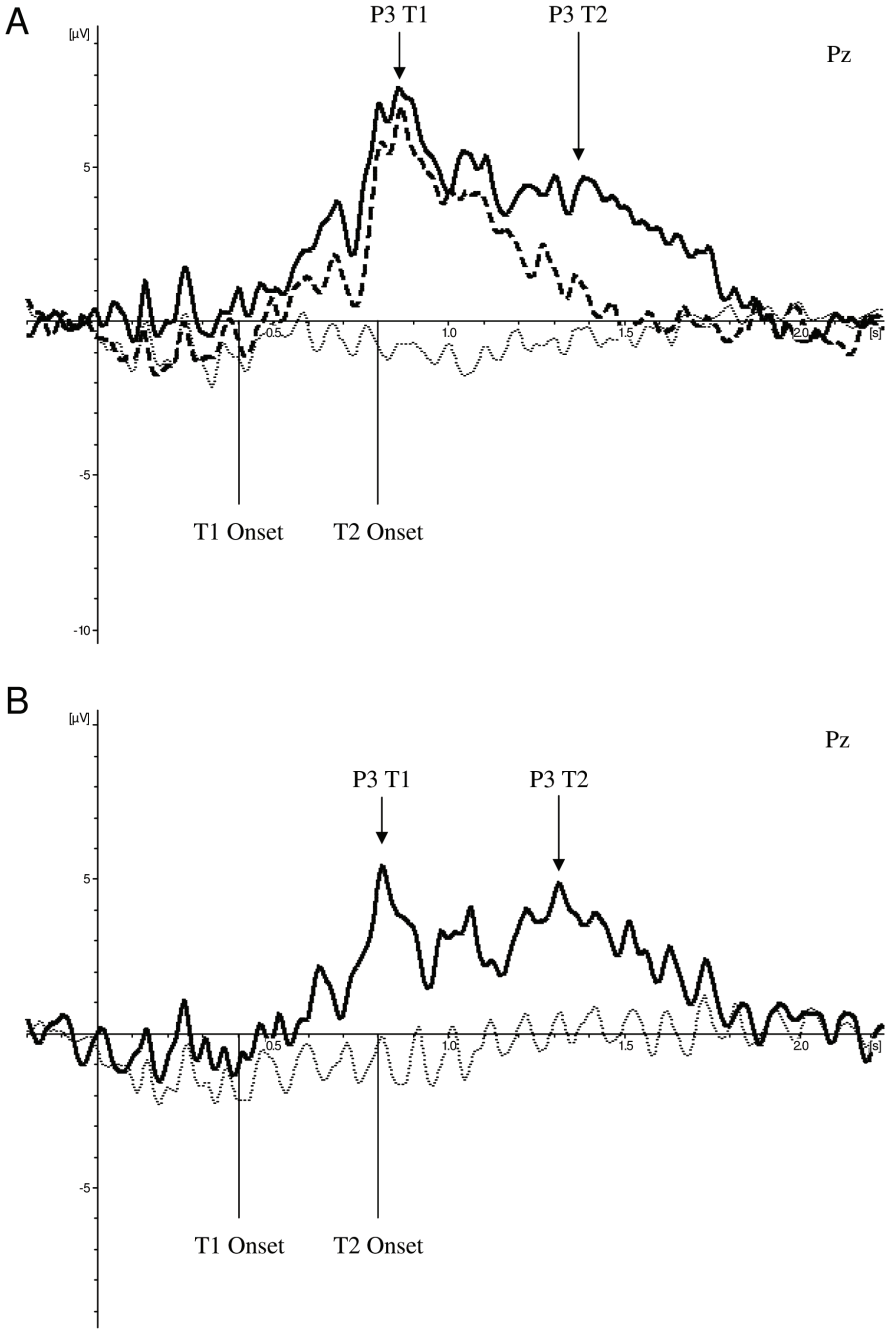
Parietal activity during blink, no-blink, and no-target trials. Grand averages of the mean activation at Pz in the standard stimuli condition of blinkers (A) and non-blinkers (B) as a function of time for SOA 400 trials during which an AB did not occur (no-blink trials, solid line), SOA 400 trials during which an AB did occur (blink trials, dashed line), and trials during which no targets were presented (no-target trials, thin dotted line). ERPs were time-locked to the onset of the RSVP stream.

A well-known hallmark of the AB is that targets that are successfully identified induce a P3 (which is typically maximal at electrode Pz) whereas no P3 is typically found for a blinked T2 [20], [28], [31]. Figure 5A shows the ERPs for blinkers in the standard stimuli condition on no-target trials, no-blink trials (i.e., trials in the SOA 400 condition in which both T1 and T2 were correctly identified), and blink trials (i.e., trials in the SOA 400 condition in which T1 was correctly identified and T2 was not correctly identified), respectively. Visual inspection of Figure 5A shows a lack of a P3 in no-target trials, and a clear T1-related P3 response in both blink and no-blink trials, consistent with the idea that the P3 reflects target consolidation, in this case of T1. In addition, a T2-related P3 response was present in no-blink trials and was absent in blink trials, which is in line with previous findings [20], [28], [31].

Figure 5B shows the ERPs for non-blinkers on no-target trials and no-blink trials. Blink trials are not presented because, by definition, non-blinkers rarely show an AB, making a meaningful analysis of these results impossible. On no-blink trials two P3 peaks can be distinguished, induced by T1 and T2, respectively, whereas no P3 component was present in the no-target trials.

#### Distractor-related mean EEG activity

**Figure 6:**
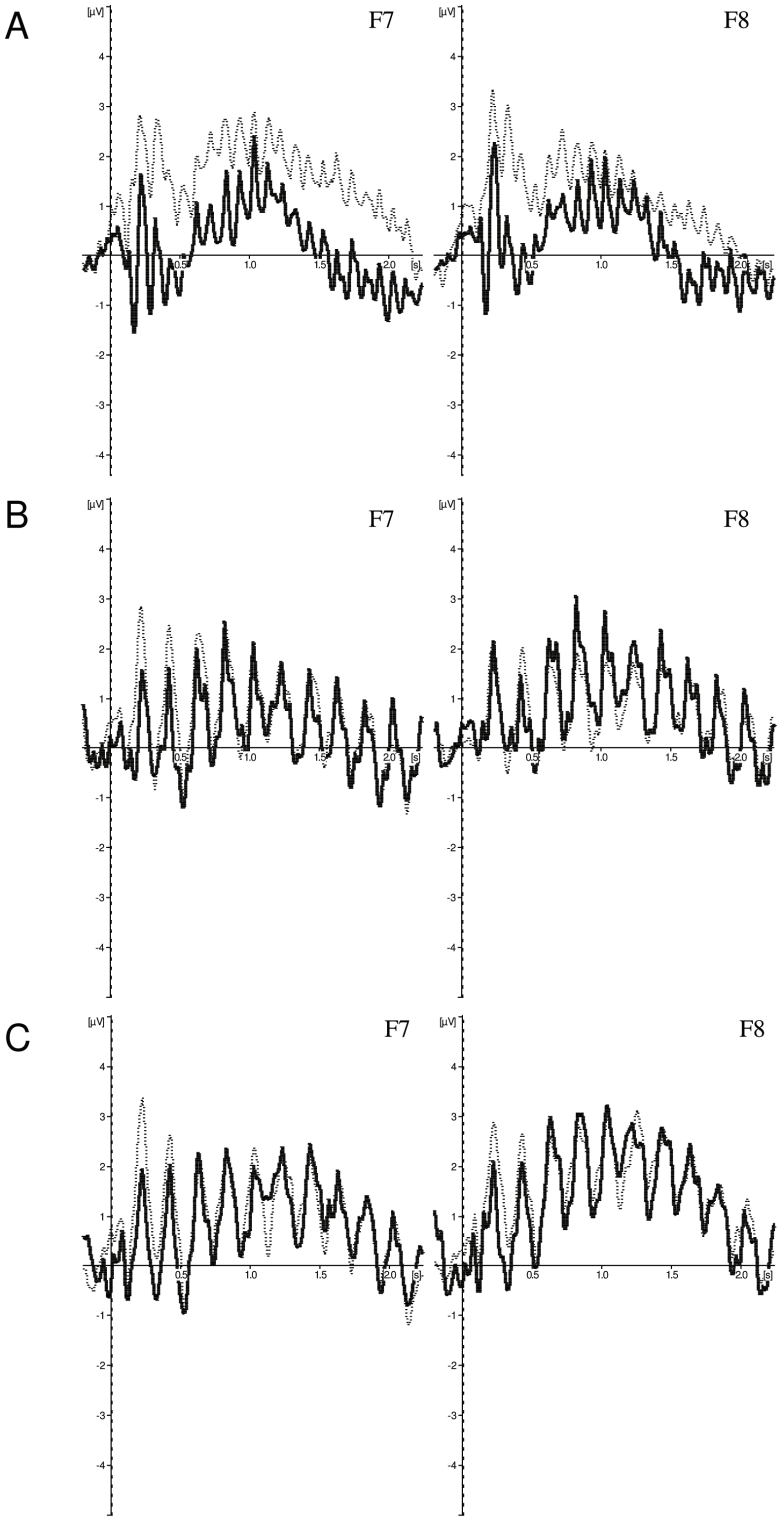
Frontal distractor-related activity. Grand averages of the mean activation at F7 (left panel) and F8 (right panel) of non-blinkers (solid line) and blinkers (dotted line) in (A) the standard stimuli condition, (B) the rotated targets condition, and (C) the rotated distractors condition as a function of time for no-target trials. ERPs were time locked to the onset of the RSVP stream.

Support for the hypothesis that non-blinkers are more efficient than blinkers in selecting targets from distractors in the standard stimuli condition but not in the letters-only conditions is provided by analyses of the no-target trials. Figure 6 shows the ERPs of trials during which only distractors were presented for electrodes F7 (left panel) and F8 (right panel) for non-blinkers (solid line) and blinkers (dotted line) in (A) the standard stimuli, (B) rotated targets, and (C) rotated distractor condition. In the standard stimuli condition, non-blinkers seemed to show less distractor-related EEG activity than blinkers did at the electrodes located above the lateral prefrontal cortex (F7 and F8) [20], [32], [33]. Independent samples t-tests conducted on the mean activity during the presentation of the RSVP stream (i.e., the mean amplitude over the entire ERP segment) showed a significant difference between non-blinkers and blinkers for electrode F7 in the standard stimuli condition, t(17) = 2.78, SE = .49, p = .017 (two-tailed), but not for F8 (p>.15). As expected, no significant differences between non-blinkers and blinkers were found in the rotated targets or rotated distractors condition (ps>.66).

#### The P3 induced by T1

**Figure 7:**
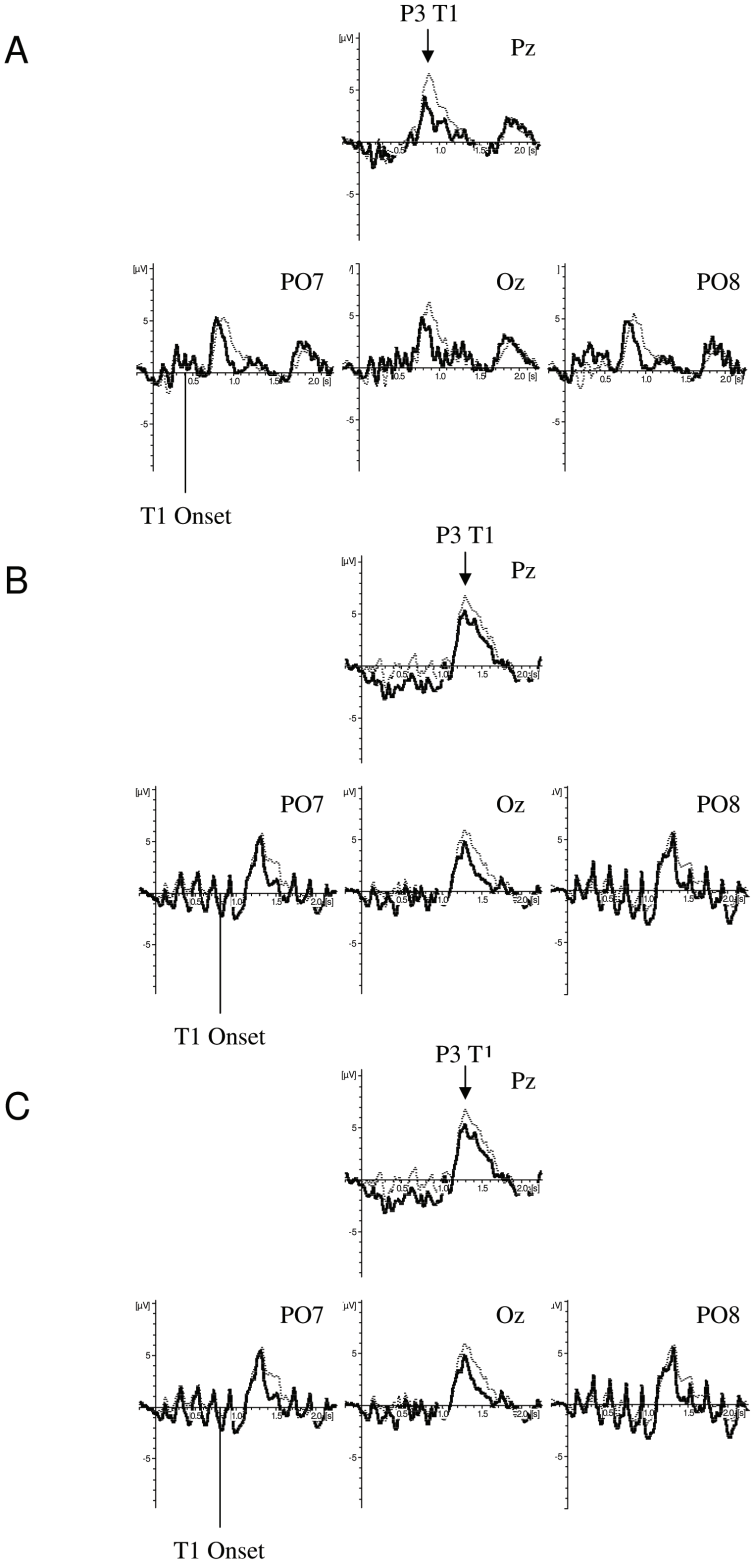
T1-related activity in each condition. Grand averages of the mean activation at Pz (middle), PO7 (bottom left), Oz (bottom middle), and PO8 (bottom right) of non-blinkers (solid line) and blinkers (dotted line) in (A) the standard stimuli condition, (B) the rotated targets condition, and (C) the rotated distractors condition as a function of time for T1-correct trials. ERPs were time locked to the onset of the RSVP stream.

In a previous study, we found that the peak latency of the P3 induced by successfully identified targets is shorter for non-blinkers than for blinkers [20]. To obtain most power, in this study, we restricted analyses to the P3 induced by T1, and determined the mean peak amplitude and latency for each individual from both single- and dual-target trials in which T1 was successfully identified. Figure 7 shows the ERPs of such trials for electrodes Pz, PO7, Oz, and PO8 for non-blinkers (solid line) and blinkers (dotted line) in (A) the standard stimuli, (B) rotated targets, and (C) rotated distractor condition. As the rate of presentation was different in the standard stimuli condition than in the rotated targets and rotated distractors condition, separate analyses were carried out.

For the standard stimuli condition, an independent samples t-tests showed a significant difference in peak latency between the T1-induced P3 at Pz in non-blinkers (399 ms) and blinkers (497 ms), t(17) = 2.81, SE = 34.90, p = .012. A repeated measures ANOVA on the peak latency of Pz, PO7, Oz, and PO8 in non-blinkers and blinkers also showed a significant effect of group, F(1, 17) = 15.16, MSE  = 6336.38, p = .001, η^2^_p_ = .47, suggesting the peak latency difference to be consistent across parietal and occipital electrodes (385 ms for non-blinkers versus 456 ms for blinkers). Although inspection of Figure 7A also suggests non-blinkers to have a smaller peak amplitude than blinkers, no significant difference in amplitude was found, neither for Pz (p>.20), nor for the other electrodes (ps>.42).

For the rotated targets and rotated distractors condition, no significant differences between non-blinkers and blinkers were found in latency (ps>.12) or amplitude (ps>.36) for electrode Pz using independent samples t-tests. A repeated measures ANOVA on peak latency with group (non-blinkers or blinkers) as a between-subjects factor and condition (rotated targets or rotated distractors) and electrode (Pz, PO7, Oz, or PO8) as within-subject factors only revealed a significant main effect of condition, F(1, 17) = 6.19, MSE  = 4725.43, p = .024, η^2^_p_ = .27, reflecting the mean latency to be shorter in the rotated targets condition (480 ms) than in the rotated distractors condition (508 ms). Importantly, neither a group effect (p>.33), nor any interactions with group were found significant (ps>.12). The same analysis was conducted on the peak amplitudes but no significant effects were found (ps>.26).

### General Discussion

A central goal of the current study was to determine whether non-blinkers avoid the occurrence of an AB by an efficient target selection process prior to working memory consolidation. The hypothesis that we tested in the first two experiments was whether such a selection process might be hindered by rotation, or that it might be based on the presence of category information.

When targets and distractors were drawn from the same stimulus category (letters) and could only be distinguished on the basis of rotation, a strong AB was found for blinkers, as well as for non-blinkers (Experiment 1). In Experiment 2, targets and distractors differed not only in rotation, but also in category (with targets consisting of letters, and distractors consisting of digits), which enabled non-blinkers to avoid the AB. Apparently, the presence of alphanumeric category information plays a critical role for the non-blinkers.

Presumably, using this category information, a shallow level of processing is sufficient for non-blinkers to select one or more targets at an early stage, mostly restricting further processing to targets only. In contrast, blinkers may be unable or at least be less efficient in making such a pre-selection, allowing for more competition and interference between stimuli at a later stage of processing, reflected in the frequent occurrence of an AB.

Given these as well as previous findings [20], [24], we predicted that non-blinkers should only show reduced distractor-related ERP activity (compared to that of blinkers) when alphanumeric category information is present, allowing them to efficiently distinguish targets from distractors. In Experiment 3, we replicated the behavioral findings from Experiment 1, and indeed found significant differences between non-blinkers and blinkers in frontal distractor-related brain activity when letter targets and digit distractors were presented, but not when targets and distractors were defined by rotation and consisted of letters only. In addition to these differences in distractor-related brain activity, we also found earlier latencies for non-blinkers' target-related activity over parietal and occipital brain areas, which is in line with findings from Martens, Munneke et al. [20]. In contrast, when stimuli consisted of letters only, such differences between non-blinkers and blinkers were no longer observed. Presumably, when category information is absent, targets and distractors are harder to distinguish, and non-blinkers are forced to process each stimulus much more elaborately, rendering an early selection of targets difficult or impossible, as reflected in the current behavioral and electrophysiological results.

#### Category-based early selection

Numerous studies on visual search have revealed that searching for a target from one category is more efficient when the target (e.g., a letter) occurs among distractors from another category (e.g., digits) than when it occurs among distractors from its own category (letters). According to Hamilton et al. [34], this alphanumeric category effect is interesting for two reasons. First, it may indicate a dissociation in the cognitive architecture between perception of digits and perception of letters, and suggest that they rely on partially independent mechanisms. Second, it suggests that learned distinctions between stimulus classes can have effects at preattentive levels of vision. Although both these points are controversial, there is compelling evidence that the effect indeed arises because letters and digit recognition depend on different cognitive mechanisms, rather than that the effect is due to perceptual differences between letters and digits [34], [35], [36]. Most interesting for the current paper are findings that the alphanumeric category effect can influence visual selection at an early stage in the processing pathway [37], which fit with our hypothesis that non-blinkers avoid the occurrence of an AB by an early target selection process prior to working memory consolidation. In contrast to a selection criterion that is based on rotation, alphanumeric category in particular seems to be a highly effective selection cue for the non-blinkers. Of course, blinkers should also be able to judge the category of a stimulus at an early processing stage, but non-blinkers appear to use this information more efficiently and effectively, at least under the current experimental conditions, such that an AB is avoided. That is, by effectively ignoring digit distractors at an early stage of processing (reflected in the reduced amount of distactor-related activity during no-target trials shown in Figure 6A), the amount of distractor interference on target processing/consolidation might be minimized. This may have reduced the ‘need’ for inhibitory processes that are meant to protect target consolidation processes but actually cause the occurrence of an AB [11], [16]. If, however, alphanumeric category information is unavailable, thereby rendering the distinction between targets and distractors more difficult, even non-blinkers are likely to ‘blink’. Indeed, in the latter case, the amount of distractor-related brain activity did not differ between blinkers and non-blinkers (see Figures 6A and B).

#### Rotation-based late selection

Many studies have found that in mental rotation tasks identification of alphanumeric stimuli occurs before mentally rotating the stimulus to determine whether it is a normal or a mirror image of the letter. If the rotation process is not necessary to arrive at a correct response, as in letter-digit discrimination of rotated alphanumeric stimuli, it is not executed and has minor or no effects on performance and electrophysiological measures [38], [39]. Experiment 2 replicates this finding in an RSVP task, supporting the idea that non-blinkers are better in selection on the basis of alphanumeric category than blinkers are.

The finding that rotation of the targets in Experiment 2 barely affected the AB is perhaps surprising given that rotation of only T1 (rather than both targets) has been found to cause a substantially larger AB in an otherwise similar task [11]. Possibly, the rotation of T1 within Taatgen and colleagues' blocked design may have led to an imbalance in the allocation of attention, inducing an additional cost for T2, which is not the case when both targets are rotated (as shown in the present study).

But why is the unrotated target condition harder than the rotated target condition (for both blinkers and non-blinkers)? Intuitively it seems easier to detect and report targets in their normal orientation amidst rotated distractors than to detect rotated targets amidst unrotated distractors. Moreover, if identification precedes mental rotation, why does it matter whether targets or distractors are rotated? When rotation affects consolidation but not identification [26], [27], rotated targets (requiring consolidation) should have a larger impact on performance than rotated distractors (requiring no consolidation).

In the rotated stimuli conditions with only letters, the selection criterion for further processing and report is whether the letter is rotated or unrotated. First, this is a rather late available, high level feature of characters, making it a more difficult and time consuming selection criterion than for instance spatial frequency or color [40], [41]. Secondly, in the rotated distractors condition, the frequency of rotated letters is high, but in the rotated target condition it is very low. The results show that it is harder to select infrequent normal targets amidst rotated letters, than infrequent rotated targets amidst normal letters. This is consistent with findings by Ilan and Miller [42], who found that reaction time to low-frequent normal characters amidst high-frequent rotated characters is longer than to normal characters amidst only normal characters. Clever experimentation suggested that this effect is the result of increased readiness for rotated stimuli, which interferes with response selection processes. In an RSVP task this increased readiness may interfere with the selection and consolidation of unrotated targets amidst rotated distractors. In the rotated targets condition, target selection and report would not be hindered by increased readiness for rotated stimuli.

#### Conclusion

Human performance is intrinsically variable, but despite this obvious fact, individual differences in AB magnitude have long been ignored. Here we present evidence suggesting that part of this variability may lie in the efficiency with which targets can be distinguished from non-targets at an early processing stage, possibly on the basis of perceptual features or the availability of well learned alphabetic and numeric category sets [43]. It is evident that more work needs to be done, but the current findings show that if category information is absent and target selection can only be based on information that is processed relatively late (e.g., rotation), even individuals who usually show little or no AB effect frequently fail to report the second of two targets when presented within 500 ms after the first. It seems more likely that the non-blinkers' difficulty to avoid an AB under these experimental conditions was due to a selection problem rather than a recognition problem, given that T1 performance remained high, and that increasing the speed of presentation has previously been shown to barely affect the non-blinkers' performance [20], [21]. It must be noted, though, that these so-called non-blinkers continued to outperform the blinkers across all conditions, suggesting that early-selection processes alone cannot fully explain the observed differences between these two groups.

Nevertheless, the current results shed new light on possible strategic mechanisms that may underlie individual differences in AB magnitude and provide intriguing clues as to how the temporal restrictions as reflected in the AB can be overcome. Moreover, they stress the important role of distractors in determining whether an AB occurs [10], [11], [13], [16], [18], [19], [20], [23], [24], [25], [43], [44], but see [45]. In addition, the present findings give rise to a number of new questions, including how task-specific the non-blinkers' ability is [21], and to what extent an individual's AB magnitude on one type of AB task reflects a general processing style such that it is predictive of that person's performance on another type of AB task that is equivalently difficult. Experiments are under way to address these questions. The notion that the AB might reflect a strategic rather than a structural limitation is consistent with the recent trend in which the cause of an AB is shifted from (a structural lack of) attentional resources to (strategic) attentional control [1].
